# Prevalence, trends and distribution of lifestyle cancer risk factors in Uganda: a 20-year systematic review

**DOI:** 10.1186/s12885-023-10621-y

**Published:** 2023-04-05

**Authors:** Annet Nakaganda, Immaculate Mbarusha, Angela Spencer, Lesley Patterson, Isla Gemmell, Andrew Jones, Arpana Verma

**Affiliations:** 1 Cancer Epidemiology and Clinical Trials Unit, Uganda Cancer Institute, Kampala, Uganda; 2 Department of Public Health and Manchester Academic Health Sciences Centre, University of Manchester, Manchester, UK

**Keywords:** Cancer, Lifestyle, Risk-factors, Prevalence, Trends, Surveillance, Control

## Abstract

**Background:**

Cancer is becoming an important public health problem in Uganda. Cancer control requires surveillance of lifestyle risk factors to inform targeted interventions. However, only one national Non-Communicable Disease (NCD) risk factor survey has been conducted in Uganda. This review assessed the prevalence, trends and distribution of lifestyle risk factors in Uganda.

**Methods:**

The review identified studies up to January 2019 by searching Medline, Embase, CINAL and Cochrane databases. Further literature was identified from relevant websites and journals; scanning reference lists of relevant articles; and citation searching using Google Scholar. To be eligible, studies had to have been conducted in Uganda, and report prevalence estimates for at least one lifestyle cancer risk factor. Narrative and systematic synthesis was used to analyse the data.

**Results:**

Twenty-four studies were included in the review. Overall, unhealthy diet (88%) was the most prevalent lifestyle risk factor for both males and females. This was followed by harmful use of alcohol (range of 14.3% to 26%) for men, and being overweight (range of 9% to 24%) for women. Tobacco use (range of 0.8% to 10.1%) and physical inactivity (range of 3.7% to 4.9%) were shown to be relatively less prevalent in Uganda. Tobacco use and harmful use of alcohol were more common in males and more prevalent in Northern region, while being overweight (BMI > 25 kg/m2) and physical inactivity were more common in females and more prevalent in Central region. Tobacco use was more prevalent among the rural populations compared to urban, while physical inactivity and being overweight were more common in urban than in rural settings. Tobacco use has decreased overtime, while being overweight increased in all regions and for both sexes.

**Conclusion:**

There is limited data about lifestyle risk factors in Uganda. Apart from tobacco use, other lifestyle risk factors seem to be increasing and there is variation in the prevalence of lifestyle risk factors among the different populations in Uganda. Prevention of lifestyle cancer risk factors requires targeted interventions and a multi-sectoral approach. Most importantly, improving the availability, measurement and comparability of cancer risk factor data should be a top priority for future research in Uganda and other low-resource settings.

**Supplementary Information:**

The online version contains supplementary material available at 10.1186/s12885-023-10621-y.

## Background

Cancer is among the leading causes of death worldwide [1–3]. Globally, there were approximately 18 million new cancer cases and 9.6 million cancer deaths in 2018 [3]. The number of new cancer cases is projected to increase to about 21 million by 2030, resulting in 17 million cancer deaths [2, 4, 5]. This increase will be relatively greater (a 93% increase) in low-income countries like Uganda [6, 7]. Evidence demonstrates that cancer is a major public health problem in Uganda and annual incidence is rapidly increasing in some areas such as Kampala, the capital city [8].

Surveillance of cancer risk factors is a powerful platform for directing cancer control strategies in any population. The World Health Organisation (WHO) recommends surveillance of at least five lifestyle risk factors: tobacco use; harmful use of alcohol; unhealthy diet; physical inactivity; and overweight/obesity [9–11]. These lifestyle risk factors are of interest because of their maximum impact on mortality and morbidity associated with cancer and other Non-Communicable Diseases (NCDs) [11]. In addition, modification of these factors is possible through effective primary prevention, and their measurement is feasible, reliable and can be obtained using acceptable methodologies [10, 12].

Although surveillance of lifestyle risk factors is vital for cancer control, only one national NCD risk factor survey has been conducted in Uganda so far, by the Ministry of Health (MOH). The MOH survey indicates that 15% of the adult population are overweight, with higher prevalence in females (19.5%) than in males (9.5%) [13]. This survey also shows that 10% of the Uganda population are current smokers, prevalence being higher in males (17%) than in females (3%) [13]. Alcohol consumption was estimated at 28.9%, with a higher prevalence in males (40%) than in females (18%) [13–15]. The MOH survey provides important information on lifestyle cancer risk factors; however, it does not show subtle changes over time and only focuses on a limited set of variables and populations [13, 16–21]. In addition, the reported information is restricted to the whole population and does not provide disaggregated data for specific regions, to guide targeted cancer control strategies [13, 15, 16, 22]. Hence, there is a need for a comprehensive and comparable cancer risk factor information at national, regional and sub-population levels; to assess progress towards implemented interventions as well as for planning and prioritizing future actions for reducing population exposure to these cancer risk factors [9, 12, 23, 24].

Therefore, this review synthesises the available evidence, to examine the prevalence, trends and distribution of lifestyle cancer risk factors among different populations in Uganda. The review also analyses structural factors (age, gender, socioeconomic etc.) that may increase the prevalence of cancer risk factors in Uganda. This provides evidence that will inform the planning and implementation of cancer control strategies and policies and direct future research on this subject.

## Methods

This systematic review followed the Preferred Reporting Items for Systematic Reviews and Meta-Analysis (PRISMA) statement [25, 26]. A review protocol was developed and registered with PROSPERO; registration number: CRD42018115265 (https://www.crd.york.ac.uk/prospero/display_record.php?ID=CRD42018115265) [27].

### Search and inclusion criteria

The review identified eligible studies, up to January 2019, by searching Medline, Embase, CINAL and Cochrane databases. Search terms for lifestyle risk factors included: smoking; alcohol use; unhealthy diet; physical inactivity; and being overweight and obesity. These search terms were combined with: Africa, East Africa; sub-Saharan Africa; Uganda, and all names of major towns and cities in Uganda. The search strategy was developed, using database-controlled thesaurus terms (MeSH for Medline and EmTree for Embase), index terms and free text terms. Search terms were combined, using Boolean operators (OR and AND) and applying truncations, wildcard and proximity operators to free text terms. EndNote reference manager was used to store the search results and to identify duplicate studies. Further published and unpublished evidence was obtained through searching relevant websites and journals; scanning reference lists of relevant studies; and citation searching using Google Scholar.

### Inclusion criteria and data abstraction

The review included studies conducted at population level in Uganda. Studies were selected if conducted in people aged fifteen years and above, and reported prevalence estimates for at least one of the lifestyle risk factors. The review excluded general discussion papers, editorials, and conference abstracts without corresponding reports. No language limitations were set. Multiple reports and papers from one study were treated as a single study and reference made to all publications. In case of discrepancies among multiple papers, information from the parent study was prioritized. In cases of significant disparities, authors were contacted for clarification.

Two reviewers (AN and IM) independently selected the studies, by screening titles, abstracts and full papers, based on pre-defined inclusion criteria. The reviewers resolved disagreements over the inclusion of studies through discussion and consensus. They also abstracted the data and appraised the quality of studies. A Data Extraction Form and excel sheet were used to extract data from the eligible studies. The review extracted information about the study setting and context; population characteristics (age, sex, region and settlement patterns); methods; lifestyle measures; study outcomes; and results. Studies were assessed for quality by highlighting their strengths and weaknesses. The quality appraisal focused on the methodology, validity, accuracy and generalizability of the results. The Critical Skills Appraisal Programme (CASP) and the Effective Public Health Practice Project (EPHPP) quality assessment tools were adapted into a 10 point criterion that was used to assess the quality of the studies [28, 29].

### Data synthesis and analysis

Descriptive data analysis was undertaken, relying on the reported results of the studies. The review tabulated and aggregated data by: lifestyle risk factors; settlement patterns (rural and urban setting); geographical region; and by sex (male and female). Studies were also tabulated by year of data collection, to assess time trends in prevalence of lifestyle factors. Time trend analysis excluded studies that did not report dates of data collection. The analysis used summary statistics (proportions and odds ratios) to compare prevalence of lifestyle risk factors among sub-populations such as: male-to-female; urban-to-rural; educated-to-non educated; and over time. For studies that reported proportions of healthy lifestyles only, prevalence proportions for unhealthy lifestyles were obtained by calculating the percentage difference between proportions. Also proportions were calculated for studies that reported absolute numbers only, using the sample size as the denominator. For studies that reported proportions for sub-regions, such as Central 1 and Central 2, an average proportion was calculated from the given proportions for the region.

## Results 

### Study selection

**Fig. 1:**
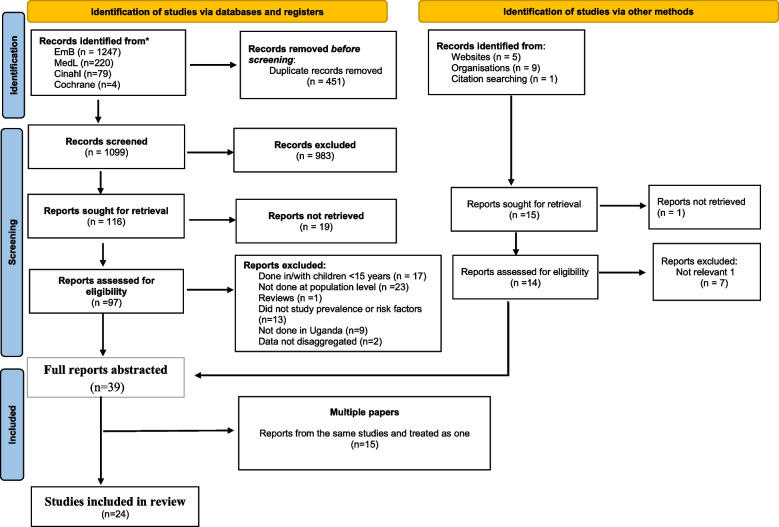
Study Selection Process: PRISMA Flow Diagram

The search of electronic databases yielded a total of 1550 articles. Of the 1550 articles, 451 articles were removed as duplicates, on review of titles and abstracts, and 983 were articles excluded as irrelevant. 116 studies remained for the full text review that involved full examination of manuscripts in detail. Eighty-four (84) studies, which did not fulfil the eligibility criteria, were excluded at this stage and the reasons documented. Eventually 32 studies were found to be eligible. Scanning the references of eligible studies, searching for grey literature and citation searching yielded a further 7 eligible articles, giving a total of 39 eligible studies. During the data abstraction process, fifteen of these 39 eligible studies were found to be multiple studies and were therefore treated as one study (six papers from MOH 2014; one paper from UBOS 2018; three from UBOS 2011; two from UBOS 2006; one from UBOS 2000–2001; and two from Germert 2016). Finally, 24 studies were included in the review. The selection process is detailed in Fig. 1 below.

### Characteristics of the studies included in the review

#### Samples and study population

Studies covered all geographical regions of the country: Northern (*n* = 2), Eastern (*n* = 6), Central (*n* = 7), Western (*n* = 6), and whole country (*n* = 7). These studies were conducted during the period 2000 to 2019; six studies conducted in the decade of 2000–2009 and nineteen studies in 2010–2019. The studies used varying sample sizes from 100 participants in Kikafunda (2005) to 65,544 participants in Kwarisiima (2016) [30, 31]. Study samples were drawn from both rural and urban populations: fifteen studies included both urban and rural participants; seven included only rural participants; and two studied only urban participants. The age of the participants studied was 15 years and above.

### Study design, outcomes and data collection

All included studies used a cross-sectional design and reported data on one or more lifestyle risk factors: tobacco use, harmful use of alcohol, unhealthy diet, physical inactivity and overweight/obesity. Nineteen out of the twenty four studies collected data using both self-administered questionnaires and physical measurements and five studies used questionnaires only. The response was above 90% among fifteen studies; 70–90% in three studies; and unreported in five studies. Appendix 1 provides details of the characteristics of the studies included in the review.

### Tobacco use

Prevalence of tobacco use was meaningfully assessed and reported in sixteen studies. The studies that assessed tobacco use are tabulated in Appendix 2, by year of field work and measures used. These studies defined and measured tobacco use using four major categories: current tobacco use, in all forms and products (*n* = 7); current smoking (*n* = 7); daily smoking (*n* = 1); and ever smoked (*n* = 1). To meet the objectives of the review, the analysis stratified and reported data by sex, settlement pattern (rural/urban), geographical region, education or social economic status and year of data collection.

**Fig. 2:**
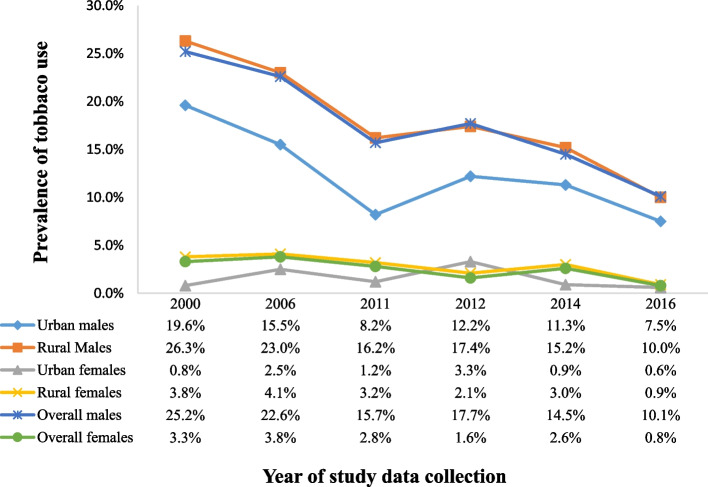
Trends of tobacco use by sex, rural, urban and overall populations in Uganda

The results indicate that national trends of tobacco use have been decreasing over time, for both sexes from 25.2% and 3.3% in 2000 to 10.1% and 0.8% in 2016 for males and females respectively [16, 19]. Tobacco use was reported to be higher in males than females, among all studies, (*P* < 0.001), using Pearson chi-square test and Logistic regression [16, 22, 32–34]. According to the most recent national study by UBOS (2018), the current prevalence range from 5.0% to 17.5% in males, compared to 0.3% to 1.1% in females. Tobacco use was also more prevalent among rural populations compared to urban: 10.0% in males and 0.9% in women versus 7.5% and 0.6% respectively, see Fig. 2 [13, 16–19, 21, 22].

These studies also indicated that tobacco use increased with age, lower education and lower sociooeconomic status (SES). For example, in the DHS 2016 survey, 1% of men aged 15–19 years smoked tobacco, compared to 22% of those aged 40-44 years; and 20% of those aged 45–49 years (data not shown) [13, 16–19]. Tobacco use was also greater in less educated groups: 2.2% in females and 24% in males with no education, compared to 0.1% females and 3% males with more than secondary level education [16]. Similarly, there were higher proportions of tobacco use among groups of lower SES: 1.2% in females and 15.2% in males in the lowest wealth quintile, compared to 0.3% females and 5.5% males in the highest wealth quintile (data not shown) [16].

**Fig. 3:**
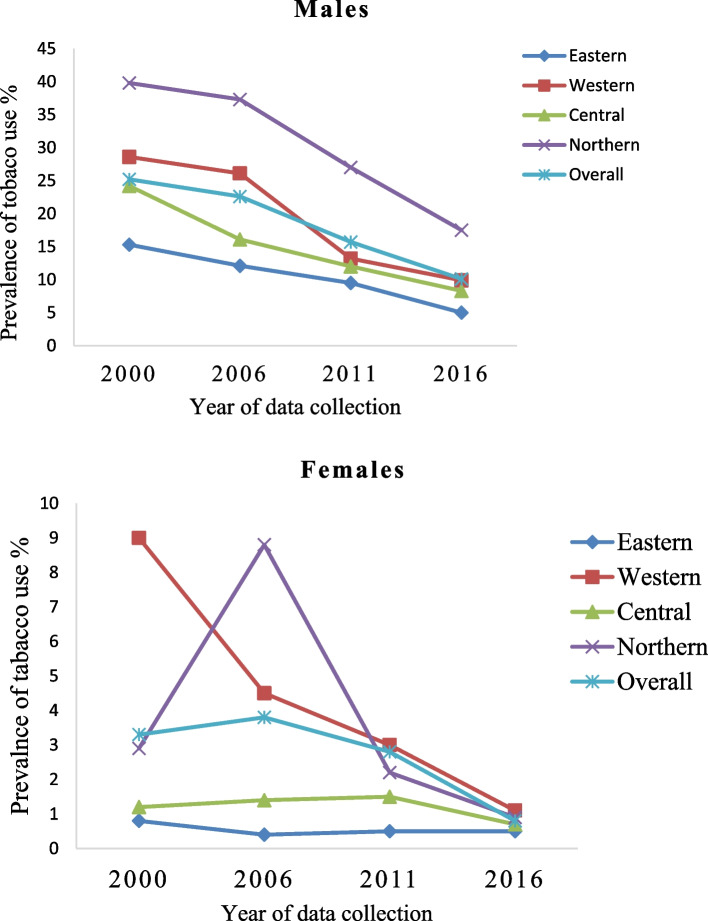
Prevalence and trends in tobacco use among Geo-political regions in Uganda

There was a decreasing trend in tobacco use across all the regions and populations over time [16–19]. However, the four regions exhibited wide variations in the prevalence of tobacco use, Fig. 3. According to the latest figures by UBOS 2018, tobacco use in males was most common in Northern region (17.5%); followed by Western (9.9%); Central (8.3%) and Eastern (5%) regions. Among females, Western region had the highest proportions of tobacco use (1.1%); followed by Northern (0.9%); Central (0.7%) and Eastern (0.5%) regions. Overall, 35% of people reported being exposed to second hand smoking at home and 43% at workplaces (data not shown) [13]. Among males, 9.8% used smoked tobacco (including cigarettes & pipe), while 1.1% used smokeless tobacco (chewing, snuff). In females, 0.9% use smoked tobacco and 0.8% used smokeless tobacco (data not shown) [16]. According to MOH 2016, 8% of the population were daily smokers and 51% of the daily smokers smoked more than five cigarettes per day (Data not shown) [13, 16].

### Factors associated with tobacco use in Uganda

**Fig. 4:**
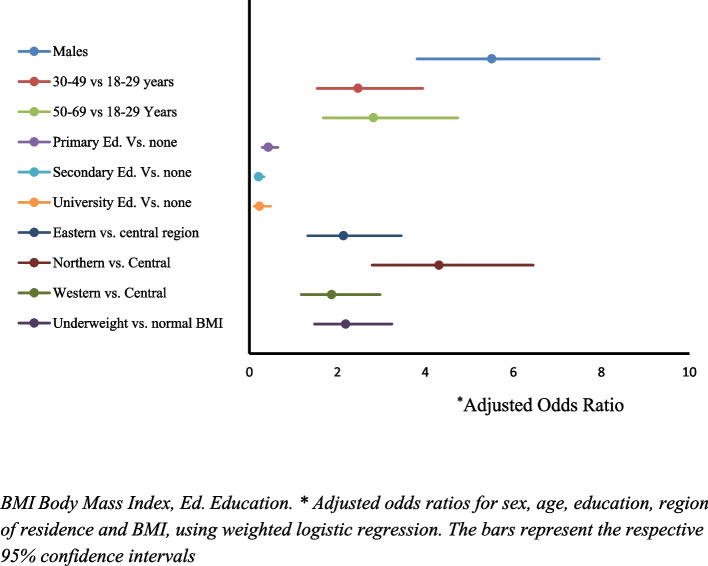
Factors associated with tobacco use in Uganda *BMI* Body Mass Index, *Ed*. Education. * Adjusted odds ratios for sex, age, education, region of residence and BMI, using weighted logistic regression. The bars represent the respective 95% confidence intervals

Out of the sixteen studies that examined tobacco use, only one reported on factors associated with tobacco use. Kabwana 2016, a multiple study from the MOH 2016, reported that age, sex, education, geographical region, and body weight were important predictors of tobacco use in Uganda, Fig. 4 [13, 21]. This study reported higher rates of tobacco use with increasing age: compared with the 18–29-year age groups, those aged 30–49 years were more likely to use tobacco, adjusted Odds ratio (AOR) 2.47, 95% CI 1.54–3.94; as were those aged 50–69 years, AOR 2.82, 95% 1.68–4.74. Males were five times more likely to be daily tobacco users, AOR 5.51, 95% 3.81–7.95 compared to females. The study also reported that, less educated individuals were more likely to be tobacco users, compared to those with higher education: AOR of 0.43, 95% CI 0.29–0.65 for individuals with primary education, compared to AOR of 0.23, 95% CI 0.11–0.48 for those with university education. In addition, residing in Eastern (AOR 2.14, 95% CI 1.33–3.45), Northern (AOR 4.31, 95% CI 2.79–6.45)) and Western (AOR 1.87, 95% CI 1.18–2.97) regions was significantly associated with daily tobacco use, compared to Central region. Being underweight was also associated with daily tobacco use, compared with people with normal body weight AOR 2.19, 95% CI 1.48–3.24 [21].

### Harmful Alcohol use

The prevalence of harmful use of alcohol was meaningfully reported in 11 (45%) studies. These studies used a range of measurements and tools to assess harmful use of alcohol including; consumption of alcohol in the past 30 days (*n* = 1) [19]; current alcohol users (*n* = 2) [34, 35]; Alcohol Use Disorder Identification (AUDIT) Test score of ≥ 8 (*n* = 3) [15, 22, 36]; WHO criteria, consumption of ≥ 5 standard units per day for men (and ≥ 4 for women) for three or more days per week (n = 4) [13, 20, 32, 33, 37]; and consumption of/or equivalent of at least 60 g of pure alcohol in the preceding month of the survey (*n* = 1) [38, 39]. A standard drink was taken to contain at least 10 g of pure alcohol by the MOH 2016 survey [13].

The review assessed harmful use of alcohol, defined as the proportion of drinkers consuming (on average) a minimum of 10 g of ethanol per day, as this is proven to be carcinogenic and to significantly increase cancer incidence rates [40, 41]. The prevalence and patterns of harmful use of alcohol are summarised in Appendix 3, by year of field work and measurements/tools used. According to the most recent national study conducted in 2014 by the MOH, the overall prevalence of harmful use of alcohol in Uganda was 18%; 26% among males and 14.3% among females [13, 20]. This study reported that, 3% are high-end drinkers, consuming about 60 g or more of pure alcohol per occasion among men and about 40 g among women. In addition, this study identified 10% of the population as having alcohol-use-related disorders [13, 20]. Trend analysis of harmful use of alcohol was not possible using the available evidence, because the only two national surveys that assessed alcohol use ( UBOS 2000 and MOH 2016) used different definitions and measurements of alcohol use [13, 19, 20].

All the seven studies that reported gender-stratified data found a significantly higher proportion of harmful use of alcohol in males, compared to females (*P* < 0.001) [13, 19, 20, 22, 32–34, 36]. Among the three studies that reported data stratified by rural and urban setting, two reported higher prevalence of harmful use of alcohol in rural males. These included UBOS (2001) who found 45.7% of rural men to be harmful users of alcohol, compared to 41.0% in urban population [19]; and MOH (2016) who found 19.6% in rural population to be harmful users of alcohol compared to 17.1% in urban populations [13, 20]. However, Kavishe 2015 found higher proportions of harmful alcohol use among urban men (13.5%), compared to rural (11.9%) [22]. A detailed analysis of the MOH 2016 NCD survey data by Kabwana (2016) found no significant difference in the prevalence of harmful use of alcohol between urban and rural residents (unadjusted *p* < 0.08) [20].

Geographically, there were higher levels of harmful use of alcohol in Northern region (23.2%), followed by Western region (21.4%), Central region (18.5%), and Eastern region (13.7%), (unadjusted *p* < 0.001) [13, 20]. Northern region also exhibited higher prevalence of alcohol use (30%) in UBOS (2001), and in Erlt (2016) (46%) [19, 36]. Erlt (2016) also identified that 89% of the alcohol consumed in Northern Uganda was homebrewed and unregulated [36].

### Factors associated with harmful use of alcohol in Uganda

**Fig. 5:**
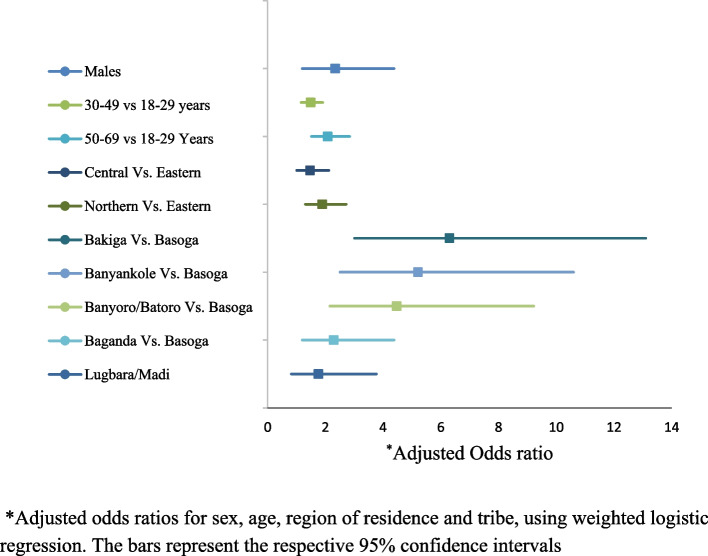
Factors associated with harmful use of alcohol in Uganda, as reported by MOH (2016), in Kabwana (2016) *Adjusted odds ratios for sex, age, region of residence and tribe, using weighted logistic regression. The bars represent the respective 95% confidence intervals

The two studies that reported on factors associated with harmful use of alcohol are: MOH (2016) in Kabwana (2016); and Nalwadda (2018) [13, 15, 20]. These studies reported that sex, age, depression, region of residence, and ethnicity were significantly associated with harmful use of alcohol, Fig. 5. Males were more likely to be medium- or high-end alcohol users with an AOR of 2.34 (95% CI = 1.88–2.91), compared to females [13, 20].

Age-specific prevalence of harmful use of alcohol increased from 13.2% among those aged 18–29 years to 25.4% in those aged 50–69 years (unadjusted *p* < 0.001): the adjusted odds ratios (AOR) were 1.49 (1.16–1.91) for those aged 30–49 years; 2.08 (1.52–2.84) for those aged 50–69-years; compared to 18–29-year age group [13, 20]. Also Nalwadda (2018) found 27.5% of men aged 54–59 years in Eastern region to be engaged in harmful use of alcohol (AUDIT score > 8), compared to 9.5% of men aged 18–28 years [15]. In addition, this study found that 83.3% of men with severe depression were harmful alcohol users, compared with 17.2% of men with no-depression [15].

Compared to Eastern region, harmful use of alcohol was more prevalent in Central region; AOR 1.47, 95% CI 1.01–2.12, and Northern region AOR 1.89 95% CI 1.31–2.72 [20]. Ethnicity was also associated with harmful use of alcohol. Compared to Basoga ethnicity, a higher prevalence of alcohol use was identified among the Bakiga ethnic group (AOR 6.30 95% CI 3.01–13.1); Banyankole, AOR of 5.21 (95% CI 2.55–10.6); Banyoro/Batoro, AOR 4.47, 95% CI 2.16–9.22; Baganda, AOR 2.29, 95% CI 1.20–4.38; and Bakiga; Lugbara/ Madi, AOR 1.76, 95% CI 0.82–3.77 [13, 20].

### Unhealthy diet

Unhealthy diet refers to high intake of foods that are high in calories (with low fibre content), salt, saturated fat or sugars and lower intake of foods such as fruits, vegetables, nuts and seeds, whole grains, seafood and unprocessed meats [22, 42–44]. However, studies in this review varied widely in their definition of unhealthy diet. Among the seven studies that addressed diet, four (Mondo 2013, Nuwaha 2013, Kavishe 2015 and Twinamasiko 2018) measured unhealthy diet using two food items (fruit and vegetable intake). Three studies: UBOS (2007), MOH (2016) and Mayega (2012), considered a range of nutrients and foods including, consumption of processed foods, salt, fatty foods; fruits, vegetables, legumes, sugary food and whole grains [13, 18, 22, 33, 34, 37–39].

To enable comparisons among studies over time, prevalence of unhealthy diet has been synthesised in this review by focusing on fruit and vegetable intake as the common measure used by all included studies. The results of the prevalence of unhealthy diet measured as insufficient fruit and vegetable consumption are summarised in Appendix 4. Studies in this review used a variety of scoring measures to indicate insufficient consumption of fruits and vegetables. These are: not consuming fruit and vegetables in the past 24-h period (*n* = 1); having less than five servings of fruit and vegetables per week (*n* = 1); consuming fruit and vegetables less than seven times in the past week (*n* = 1); having fewer than one serving of fruit or vegetables per day (*n* = 1); having fewer than five servings of fruits and vegetables per day (*n* = 1); and consuming fruits and vegetables less than 3 days a week (*n* = 1). According to the WHO, an unhealthy diet includes an intake of less than 5 servings of fruit and vegetables per day [13].

According to the latest national survey conducted in 2014 by MOH (2016), 87% of females and 88% of males in Uganda consumed less than 5 servings of fruit or vegetables per day and 27% had not consumed any fruit or vegetables in the preceding week of the interview [13]. Apart from the DHS survey done by UBOS in 2006, the few studies of unhealthy diet in Uganda started in 2011 and the available evidence is not sufficient to undertake meaningful trend analysis and comparisons of unhealthy feeding practices among different populations. Nevertheless, more than half of the Ugandan population do not meet the WHO recommendations of having 5 or more servings of fruits and vegetables per day and hence have exhibited insufficient levels of fruit and vegetable consumption across all the studies [13, 18, 22, 33, 34, 37].

Among the four studies that reported gender-stratified data, three studies, Mondo (2013), Kavishe (2015) and MOH (2016), reported similar proportions of insufficient fruit/vegetable intake among males (60%-99%) and females (58%-99%) [13, 22, 33, 34]. In the two studies, UBOS (2007) and Kavishe (2015), that reported data by residence, one study (Kavishe 2015) found higher levels of insufficient fruit and vegetable intake among rural populations (64–70%) compared to urban populations (58–60%) [18, 22]. None of the studies assessed factors associated with unhealthy diet in Uganda but MOH (2016) found younger respondents to be more likely to consume fruits and the elderly more likely to consume vegetables [13].

Considering other measures of unhealthy diet, MOH (2016) identified that approximately 5% of the Ugandan population consumed processed food and 13% reported excess salt intake [13]. Previously, the DHS (2007) survey identified that the staple diet taken by mothers of young children consisted of foods made of legumes (68%) and grains (67%). This study also indicated that 30% of women consumed meat, fish, shellfish, poultry or eggs in the past 24-h period; 20% consumed milk; 5% consumed cheese or yogurt; 35% took tea or coffee; and 33% consumed foods made with oil, fat or butter [18]. Mayega (2012) used an “individual dietary diversity score” to assess food quality in the past 7 days prior to the survey and found that 90% had low-moderate dietary scores (0–6 out of 9) [38, 39].

### Physical inactivity

Eight studies reported some data on physical inactivity (PA). Appendix 5 provides the prevalence of physical inactivity and the measures used by the different studies. Apart from one study, Kirunda (2016), that used a Pedometer Watch, all studies measured physical inactivity by self-report. However, there were variations in the definition of physical inactivity across studies including sedentary occupation (*n* = 1); lack of activity at workplace, during recreation, leisure or travelling (*n* = 2); less than 3 times of physical activity (PA) like walking, riding, exercises, sports, manual work per week (*n* = 1); no vigorous activity per week (*n* = 2); less than 7500 steps taken per day (*n* = 1); and achieving less than the WHO recommendations of at least 150 min of moderate-intensity PA or; 75 min of vigorous-intensity PA or an equivalent combination of moderate and vigorous intensity PA totalling at least 600 MET-minutes (*n* = 1) per week. [45]. The WHO definition for PA combines intensity with duration of PA which was utilised by MOH (2016) [13]. None of the remaining seven studies indicated the duration or intensity of PA, so they do not comply with the WHO definition. The differences in measurements of physical inactivity prevented meaningful comparisons of physical inactivity levels among studies and among populations.

According to the latest national survey conducted in 2014, the overall prevalence of physical inactivity in Uganda (according to WHO recommendations) is 4.3%: 3.7% among men and 4.9% among females [13]. However, as shown in Appendix 5, higher prevalence of physical inactivity has been reported in four subnational studies: Mondo (2013), Kavishe (2015), Kirunda (2016) and Twinamasiko (2018) ranging from 29 to 96% among various populations of the country [22, 33, 37, 46].

Among the four studies that reported gender-stratified data, two studies, Kavishe (2015) and Kirunda (2016), found females to be significantly more physically inactive than males (*P* < 0.001) [22, 46]. However, MOH (2016) and Mondo (2013) did not find any significant difference between males and females [13, 33]. Two studies, Kavishe (2012) and MOH (2016) reported data by residence status (urban/rural) and both found higher levels of physical inactivity among urban populations compared to rural populations [13, 22]. According to the national survey by MOH (2016), Central region had higher levels of physical inactivity (8.1%) followed by Eastern (6.8%), Western (3.4%) and Northern region (3.3%) [13, 45, 47].

### Factors associated with physical inactivity

**Fig. 6:**
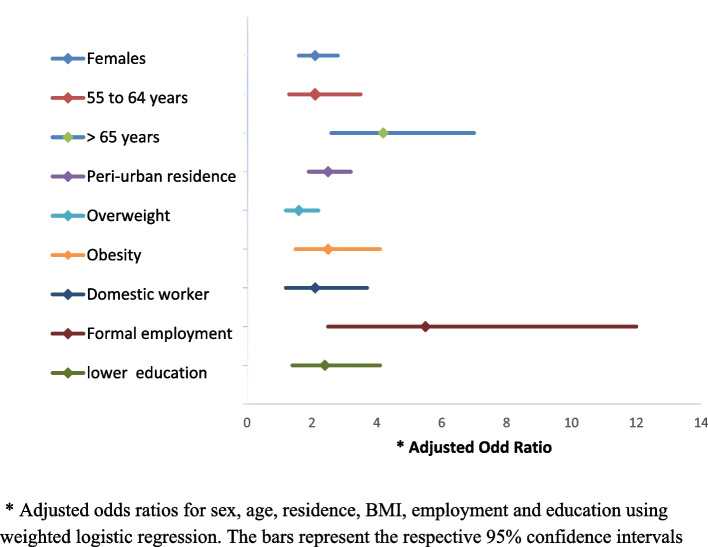
Factors associated with physical inactivity in Uganda according to MOH (2016), in Kirunda (2016) * Adjusted odds ratios for sex, age, residence, BMI, employment and education using weighted logistic regression. The bars represent the respective 95% confidence intervals

One study MOH (2016) reported in Kirunda (2016) and Guwatudde (2016) assessed factors associated with physical inactivity, Fig. 6. According to Kirunda (2016), being female; being 65 years and above; residing in an urban area; engaging in domestic work; being a student; being in formal employment; and having attained lower primary education were significantly associated with physical inactivity [13, 45, 46].

According to MOH (2016), reported in Kirunda (2016), women were 2 times more likely to be physically inactive, with AOR of 2.1 (95% CI: 1.6 to 2.8), compared to men. Physical inactivity also increased with age, with AOR of 2.1 (95% CI: 1.3 to 3.5) among people aged 55 to 64 years, and 4.2 (95% CI: 2.6 to 7.0) among those aged ≥ 65 years compared to those aged 18–29 years. In addition, residing in peri-urban areas was associated with physical inactivity, at AOR 2.5 (95% CI: 1.9 to 3.2), as was being overweight AOR of 1.6 (95% CI: 1.2 to 2.2), and obese, AOR of 2.5 (95% CI: 1.5 to 4.1) [46]. Kirunda (2013) also identified associations between sedentary behaviours (taking an average of < 5000 steps per day) and being female, AOR of 3.4 (95% CI: 2.4 to 4.9; aged ≥ 65 years AOR of 4.9 (95% CI: 2.5 to 9.7); residing in a peri-urban area AOR of 2.9 (95% CI: 1.9 to 4.6); being a domestic worker AOR of 2.1 (95% CI: 1.2 to 3.7); being in formal employment AOR of 5.5 (95% CI: 2.5 to 12.0); and having attained a lower level of education, AOR of 2.4 (95% CI: 1.4 to 4.1) [46]. Similarly, MOH 2016 (reported in Guwatudde 2016) found BMI and level of education to be associated with physical inactivity. Obese participants were less likely to meet the WHO PA recommendations with adjusted AOR of 0.92 [95% CI = 0.85 to 0.99] [13, 45]. MOH (2016) also found university and higher education to be associated with physical inactivity, AOR 0.95,95% CI = 0.90 to 0.99 when compared to no schooling at all [13, 45].

### Overweight and obesity

Being overweight is defined as body mass index (BMI) > 25 kg/m^2^ and Obesity as BMI > 30 kg/m^2^ [48]. Overweight and obesity were reported in 18 studies. All the studies used the same measures of overweight: Body Mass Index (BMI) of 25–29.9 kg/m^2^ and Obesity as BMI of 30 kg/m^2^ and above. Fifteen studies reported separated proportions for overweight and obesity while 3 studies reported aggregated data as overweight (BMI ≥ 25 kg/m^2^). Appendix 6 provides the prevalence of overweight and obesity by study.

**Fig. 7:**
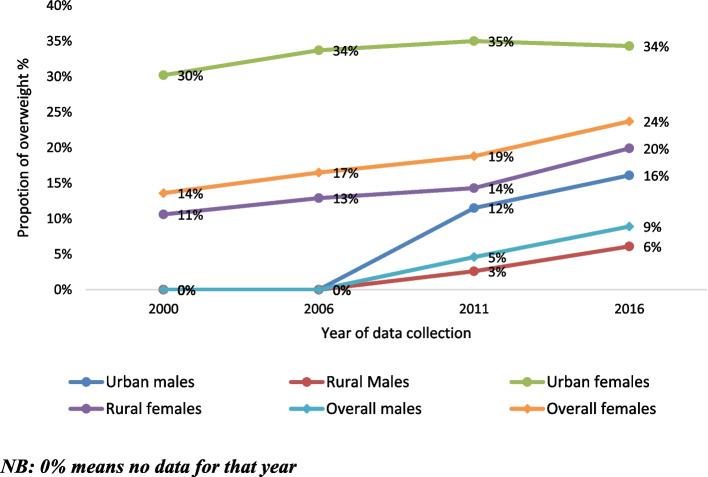
Prevalence of overweight and obesity (> 25 kg/m2) in Uganda NB: "0%" means no data for that year

**Fig. 8:**
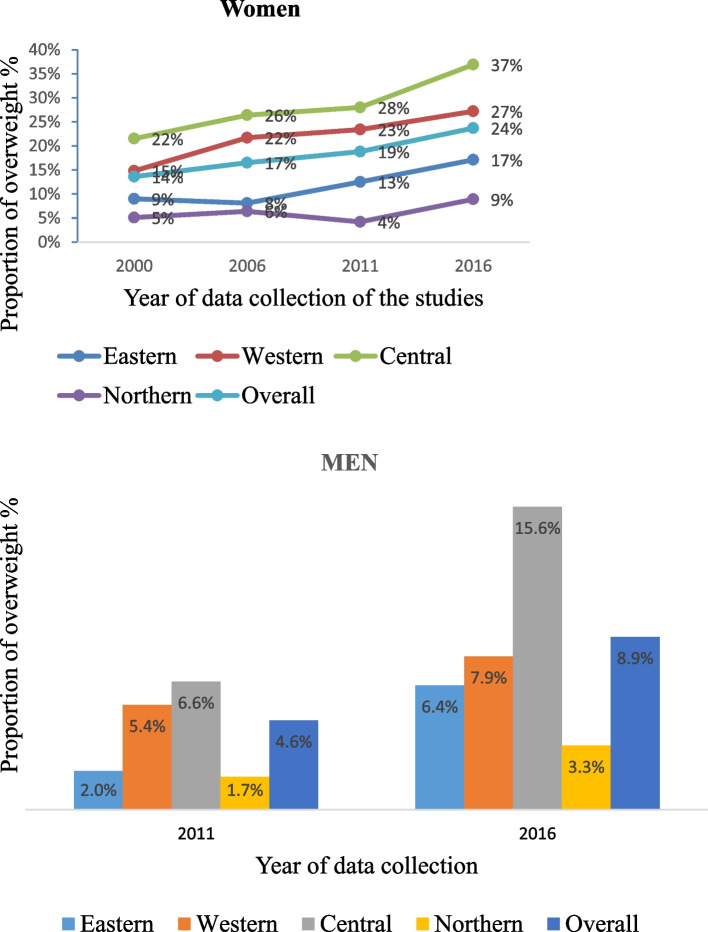
Prevalence and trends of overweight among geo-political regions in Uganda

Studies of being overweight started in 2000 by the DHS surveys in women only, with no national data on being overweight for men until 2011. According to UBOS (2018), 7.7% of men and 16.5% of women were overweight and 1.2% of males and 7.2% of women were obese in Uganda [49]. There has been a steady increase in the reported prevalence of overweight and obesity in Uganda over time (Figs. 7 and 8). The overall proportion of women who are overweight (BMI above 25 kg/m^2^) has increased from 14% in 2001 to 24% in 2016 [17–19, 49]. Similarly, the proportion of men who are overweight has increased from 5% in 2011 to 9% in 2016 [17, 49].

Twelve of the eighteen studies reported gender-stratified data and all found overweight and obesity to be more prevalent in women than men. According to the latest national survey by UBOS (2018), overweight proportions ranged from 8.5% to 22.4% in women versus 3.0% to 13.1% in men,while obesity ranged from 1.5% to 14.6% in women versus 0.5% to 3.2% in men.

Seven out of eight studies reported data by residence status (rural/urban) and all found overweight and obesity to be more prevalent in urban populations than rural populations [13, 18, 22, 50–53]. Overweight proportions ranged from 21.5% to 30.4% among urban women versus 10.5% to 30.3% among rural women; and from 13.1% to 15.8% among urban men compared to 2.3% to 8.5% among rural men [13, 18, 19, 22, 49, 50]. Similar patterns were observed for obesity with ranges of 5.6% to 23.1% among urban women versus 1.4% to 18.9% among rural women; and 1.7% to 6.3% among urban men compared to 0.3% to 2.4% among rural men, Fig. 7. Only one study, Ajayi (2016), found a higher prevalence of being overweight in rural populations (35.5% in men and 41.9% in women) than in peri-urban populations (13.7% of men and 32.7% of women) [23]. However, this same study found obesity levels to be higher in peri-urban populations (14%) compared to rural (10%) (*P* < 0.001) [23].

Geographically, prevalence of overweight and obesity is steadily increasing in all the regions of the country and were most prevalent in the Central region from 2000 to 2016(Fig. 8) [16–19]. Data from 2016 shows Central region to have the highest levels of overweight of 15.6% and 37% among men and women respectively; followed by Western region (7.9% of men and 27% of women); Eastern region (6.4% of men and 17% of women); and Northern region (3.3% of men and 9% of women) [16].

### Factors associated with overweight and obesity

Six studies reported factors associated with overweight or obesity in Uganda. Factors found to be associated with being overweight/obese were sex, urban residence, higher education, higher Social Economic Status (SES), marital status, age, history of diabetes, smoking, alcohol consumption and physical inactivity (Appendix 7) [23, 38, 50, 54–58].

Significantly, the most common factor found to be associated with being overweight and obesity was being female as determined by all the five studies that compared prevalence of being overweight between females and males [23, 39, 50, 54, 55]. Females were 2–5 times more likely to be overweight than males. Odds Ratios were found to be higher in some studies such as Ajayi (2016), where being female was associated with obesity after adjusting for age and marital status with high AOR of 11.22; 95% CI: 2.27- 55.40 [23]. Adjusted Odds Ratios were much higher in urban areas where females were found to be twenty eight times more likely to be obese [AOR = 27.80; 95% CI: 7.13, 108.41] [23]. Other risk factors commonly found to be associated with being overweight were higher Social Economic Status (SES), reported in four studies with up to five times the likelihood of being overweight than lower SES [17, 39, 50, 55–57]. Increasing age was also reported in three studies with ranges of 2 to 6 times of being overweight compared to 18–24-year age group [39, 50, 55]. Finally, residing in urban areas (also reported in 3 studies) showed more than three times the odds of being overweight compared to rural populations, Appendix 7 [17, 39, 50, 56–58].

**Fig. 9:**
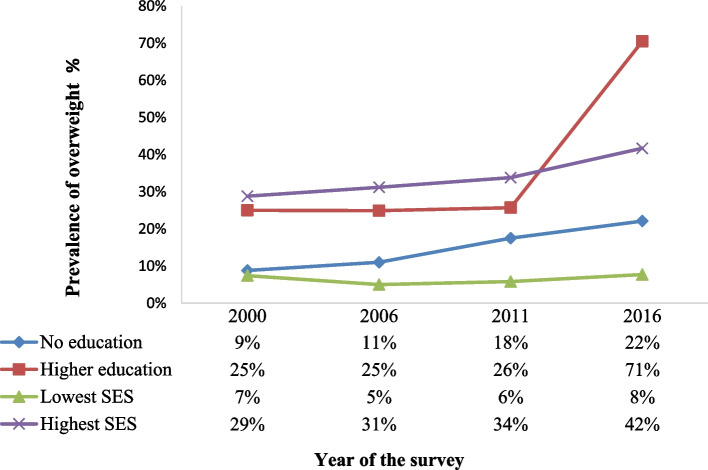
Prevalence trends of being overweight by education and wealth status among females in Uganda

UBOS demography health surveys have also reported similar predictors of overweight/obesity since 2000. For example, UBOS 2011 and 2016 surveys found the proportion of women who are overweight or obese to increase with age, from 11–12% among those aged 15–19 years to 27–34% among those aged 40–49 years [16, 17, 49]. Moreover, these surveys show that women in the Central and Western regions were more likely to be overweight than women from other regions of the country; and that being overweight increased with increasing education and wealth status, (p = 0.01) [16–19, 56, 57]. For example, in UBOS (2016), 42% of women in the highest SES were overweight or obese, compared with 8% of women in the lowest SES, and 71% of women with higher education (secondary and above) were overweight compared to 22% with no education (Fig. 9) [16, 56].

### Quality assessment of included studies 

Two reviewers (AN and IM) appraised the quality of the twenty-four studies included in the review, using a 10-point tool adopted from CASP and EPHPP tools. The appraisal of studies assessed the clarity of the study objectives; sample selection and recruitment procedures; definition of risk factors; data collection; data analysis and reporting; ethical considerations; and integrity of the study. Overall, fifteen studies (63%) were rated to be of high quality (9–10 points) and nine studies (38%) to be of moderate quality (7–8 points). All studies had a well-defined research question, with clear aims and objectives, and all used reliable and valid methods of data collection. Apart from one study, all studies had strong sampling techniques. However, the majority (23 out of 24 studies) did not report sample size calculation and five studies omitted details of non-responders. Quality of studies was also compromised by not using or adopting any internationally standardised tool for measuring cancer risk factors (in eight studies) and lack of research ethics review of the studies prior to implementation (in five studies).

## Summary and Discussion

This review was designed to provide a high level overview of lifestyle cancer risk factors in Uganda, to inform prevention strategies and direct future cancer research. Based on the available evidence, this is the first systematic review on lifestyle cancer risk factors in Uganda. These results show that surveillance data for lifestyle cancer risk factors in Uganda is rare. Up to 2019, only 24 studies had meaningfully reported on lifestyle cancer risk factors in the general population in people aged 15 years and above, despite a sensitive search strategy being applied. This paucity of data hinders rational planning and implementation of evidence-based cancer strategies in the country [24]. Nevertheless, this review shows unhealthy diet (88%) to be the most prevalent lifestyle cancer risk factor for both males and females. This is followed by harmful use of alcohol (range of 14.3% to 26%) for men and overweight/obesity (range of 9% to 24%) for women. Tobacco use (range of 0.8% to 10.1%) and physical inactivity (range of 3.7% to 4.9%) were shown to be relatively less prevalent in Uganda [12, 15].

Due to scarcity of information, comparisons among populations and trend analysis were difficult for some lifestyle risk factors, including: unhealthy feeding; physical inactivity; and harmful use of alcohol. As a result, trend analysis and regional comparisons were possible for tobacco use and overweight/obesity only. The results show a gradual decrease in tobacco use and a steady increase in the levels of overweight and obesity over time [16–19]. Although the decreasing trend of tobacco use in Uganda differs from the increasing trends observed in Sub-Saharan Africa, it is in line with the decreasing trends seen globally [59–61]. The decline in tobacco use in Uganda resonates with its sustained effort towards tobacco control. Uganda’s tobacco control efforts started in 2007 by joining the WHO Framework Convention on tobacco and later instituting a Tobacco Control Act, 2015 [62], the primary legislation regulating tobacco products and tobacco use in Uganda. This Act led to numerous actions against tobacco use, including: creating a smoke free environment and banning smoking in public places and those with children present; banning tobacco advertising, promotion and sponsorship; regulating all tobacco product sales, packaging and labelling; and protection against tobacco industry interference [63]. Hence, the observed decreasing trend of tobacco use indicates some impact of Uganda’s tobacco control efforts.

The increasing trend in prevalence of being overweight and obesity is consistent with that reported recently for many African countries. The prevalence of being overweight has doubled in the past two decades in many African countries including: Uganda, Kenya, Rwanda, Benin, Niger and Ivory Coast; and tripled in Zambia, Burkina Faso, Mali, Malawi and Tanzania [64, 65]. Similarly, this review shows very high levels of being overweight (BMI ≥ 25 kg/m^2^) in some Ugandan sub-populations, such as urban females (34%) and females in central region (37%) [16]. These observed patterns and trends, suggest that being overweight and obesity have reached epidemic levels in Uganda and there is an urgent need for interventions to improve diet and physical activity patterns in the country, to control overweight prevalence [65, 66].

Regional analysis shows prevalence of tobacco use to be highest in Northern Uganda, while overweight/obesity proportions are highest in Central region. This observation was consistently seen across all the four national demographic surveys by UBOS, which reported a higher prevalence of these risk factors in these regions since 2000, as well as the MOH (2016) survey [13, 16–19]. Several factors could explain this observation. First, tobacco is widely grown in Northern and Western regions and this could expose these communities to higher levels of tobacco use [67, 68]. Second, it is likely that these geographical patterns are a result of the differences in socioeconomic conditions and settlement patterns among regions. In Uganda, low levels of urbanisation, social-economic deprivation and education inequality remain higher in the Northern region compared to other regions, due to political conflicts, high unemployment and low economic development [69–73]. Evidence shows tobacco use to increase with lower levels of education and SES and to be greater in rural populations [13, 21, 74]. These associations are reversed for overweight and obesity, with higher levels of overweight and obesity in Central region which is more urban, with higher levels of education and social-economic status, compared to Northern region [13, 21, 70–72].

All the studies included in the review employed a cross sectional design, and factors found to be associated with the distribution of life style factors in Uganda included:

- Age: prevalence of all lifestyle risk factors increased with increasing age.
- Settlement patterns: tobacco use, harmful use of alcohol and unhealthy diet were more prevalent in rural populations compared to urban, whereas being overweight and physical inactivity were more prevalent in urban than in rural populations.
- Region of residence: tobacco use and harmful use of alcohol were more common in Northern and Western regions, while being overweight was more common in Western and Central regions.
- Education status: tobacco use and harmful use of alcohol were more prevalent in less educated groups while being overweight/obesity and physical inactivity were more prevalent in those with higher education.
- Social Economic Status: tobacco use and harmful use of alcohol were higher in people with lower SES, and being overweight/obesity and physical inactivity were higher in those with higher SES.
- Sex: being male was associated with tobacco use and harmful use of alcohol, while being female was associated with overweight/obesity and physical inactivity.

These findings are in line with the available evidence in Africa, where observational studies have identified that, being male, older age, lower education status and lower SES are associated with tobacco use and harmful use of alcohol in Africa [75, 76]. Similarly increasing age; being female; residing in urban areas; formal employment; higher education; and higher SES have been reported in several studies to be associated with being overweight, obesity and physical inactivity in African settings [65, 66, 77, 78].

Another important issue that has emerged from this review is the wide variation in the definition and measurements of lifestyle risk factors across the studies. Measurement of lifestyle factors requires uniformity and standard definitions for indicators to allow comparisons between populations over time. This calls for urgent strategies to enhance uniformity and consistency in the methods of assessing cancer risk factors in Uganda. Strategies for improving cancer risk factor data might involve establishing a comprehensive monitoring framework and developing a locally relevant standardised tool, with standard definitions for unhealthy lifestyle risk factors. There are standard definitions for lifestyle indicators that are endorsed internationally by health organisations like the WHO. The WHO provides a tool for assessing NCD risk factors (the WHOStepwise tool), and recommends countries to expand and adapt the tool to local needs and interests [11, 13] The evidence from this review strongly implies that such tools should be tailored to local context and practices [79].

Most importantly, the locally adapted standardised tools should be based on the available scientific evidence about the levels of lifestyle factors considered to be carcinogenic or proved to cause harm to human health. On review of the included studies, reasons for wide variations in the measurement of lifestyle risk factors stemmed from differences in the definition of what constitutes an unhealthy lifestyle. For example, most of the studies focused on fruits and vegetables consumption to measure unhealthy diet. However, it is currently recommended to focus on overall dietary patterns (rather than single food items) to measure unhealthy diet, using tools like the Alternative Healthy Eating Index (AHEI) and Health Diet Indicators (HDI) that consider the balance of all food groups including those recommended for daily consumption and those that are not [44, 80]. Epidemiological evidence shows that diets defined as healthy within such tools are associated with a 20% reduction in the risk of NCD related deaths including cancer [80]. Similarly, WHO defines harmful use of alcohol as “a pattern of psychoactive substance use that is causing damage to health” [81]. Many researchers may still find it challenging to measure “damage to health”, especially when there are no exhibited symptoms of harmful use of alcohol in an individual. Among the compounds of alcoholic beverages, ethanol was found to have the highest risk of causing cancer even at moderate drinking levels [41]. An average intake of 10 g of ethanol per day is proved to be carcinogenic and to significantly increase cancer incidence at population levels [40, 41]. Hence, tools should aim at estimating such content, volume, duration and frequency/pattern, when assessing harmful use of alcohol. Applying these criteria to measurement tools would permit logical assessment of risk factors at population level in Uganda.

### Limitations and strengths of the review

The principal limitation of this review is the lack of detailed information about the studied variables and the variance in methodology used by the included studies. Information on some lifestyle risk factors has not been collected over time and there were variations and changes in the definitions of these factors. This made comparisons across populations and over time very difficult. Although the findings should be interpreted with caution, this review provides the first comprehensive descriptive assessment of lifestyle cancer risk factors in Uganda. These findings contribute in several ways to our understanding of the roadmap to take to address the burden of cancer risk factors and provide a basis for future improvements in measuring lifestyle risk factors in Uganda.

## Conclusion

Overall, there is limited data about lifestyle risk factors in Uganda. Apart from tobacco use, other factors seem to be increasing in Uganda and there is variation in the prevalence of lifestyle risk factors among different populations in Uganda. The findings of this review can be used to develop targeted interventions aimed at identifying, preventing and treating these cancer risk factors at individual, regional and population levels. Preventing these risk factors requires policies that engage all the concerned sectors, since many of the observed drivers of the lifestyle risk factors fall outside the mandate of MOH. A multi-sectoral approach therefore, involving all the stakeholders such as planning and development; trade and industry; agriculture, food and nutrition; transport; education and sports; urban planning; and social media and communication, will ensure a comprehensive approach to cancer control. Above all, improving availability, measurement and comparability of risk factor data, by establishing a comprehensive monitoring framework and an evidence-based standardised tool for assessing cancer risk factors, should remain a top priority for future research in Uganda and other low-resource settings.

## Supplementary Information

**Additional file 1.**

## Data Availability

All data generated or analysed during this study are included in this published article. Since this is a systematic review of available literature, there is no raw data collected. So all the information generated and analysed is included in the manuscript and relevant tables and figures.
