# Supplementary material for: Prevalence, trends and distribution of lifestyle cancer risk factors in Uganda: a 20-year systematic review
Source: BMC Cancer. 2023 Apr 5;23:311. doi: 10.1186/s12885-023-10621-y (PMC10077672; doi:10.1186/s12885-023-10621-y)
Supplement: Supplementary file 1 — Additional file 1. [file 12885_2023_10621_MOESM1_ESM.docx]

**Appendix 1: Characteristics of included studies**

| Study characteristics | | | Population characteristics | | | | |
| --- | --- | --- | --- | --- | --- | --- | --- |
| a. Author (ref. no.) b. Date of fieldwork  c. Date of publication | Aim of the study and outcomes studied | a. Study design b. Sampling method c. Response  d. Method of data collection | Sample size (no.) | | | a. Geographical Region b. Rural/Urban c. Name of a place | a. Target population  b. Age rage |
|  |  |  | Total | Men | Women |  |  |
| a. UBOS b. 2000-2001 c. 2001 | Provide information on fertility; family planning; mortality; maternal and child health and nutrition; and knowledge related to HIV/AIDS (DHS-2000) a. Tobacco use b. Alcohol use c. overweight/obesity | a. Cross-sectional b. Stratified cluster sampling  c. 96% d. Q & M | 9208 | 1962 | 7246 | a. All regions  b. Urban and Rural c. Whole country | a. Whole country b. 15-54 years |
| a. Kikafunda  b. 2002 c. 2005 | Determine nutritional status and functional ability of the elderly.. a. overweight/obesity  b. Unhealthy Diet | a. Cross-sectional b. Stratified random sampling  c. 95.2% d. Q & M | 100 | 44 | 56 | a. Central b. Rural  c. Mpigi | a. elderly b. 60 to 90 years |
| a. Baalwa  b. 2004 c. 2010 | Prevalence of overweight and obesity and their associated factors.  a. Overweight/obesity | a. Cross-sectional b. Stratified random sampling c. NR d. Q & M | 683 | 338 | 345 | a. Central and Eastern b. Urban and Rural c. Kampala and Kamuli | a. young adults enrolled in schools in rural and urban Uganda b. 18-30 years |
| a. UBOS b. 2006 c. 2007 | Information on fertility; family Planning; infant, child, adult, and maternal mortality; maternal and child health; nutrition; and knowledge of HIV/AIDS (DHS-2006) a. Tobacco use b. Unhealthy diet c. overweight/obesity | a. Cross-sectional b. Multistage cluster sampling c. 98% d. Q & M | 11034 | 2503 | 8531 | a. All regions  b. Urban and Rural c. Whole country | a. Whole country b. 15-54 years |
| a. Wamala  b. 2006 c. 2009 | Prevalence and factors associated with hypertension a. Tobacco use b. Alcohol use c. overweight/obesity | a. cross-sectional b. Multistage random sampling c. NR d. Q & M | 842 | 441 | 401 | a. Western  b. Rural c. Rukungiri. | a. rural district of Rukungiri, Uganda b. > 20 years |
| a. Kizza b. 2008-2009 c. 2015 | Prevalence of Latent Tuberculosis infection and the associated risk factors  a. Tobacco use | a. Cross-sectional b. Multistage random sampling c. 98% d. Questionnaire | 283 | 91 | 192 | a. Central b. Urban c. Kampala | a. adults in Kampala,  b. > 15 years |
| a. Ertl  b. 2010 c. 2016 | Patterns, determinants and consequences of alcohol use disorders. a. Alcohol use | a. Cross-sectional b. NR c. NR d. Questionnaire | 669 | 304 | 365 | a. Northern b. Urban and Rural C. Gulu and Nwoya | a. Post-conflict population b. adults |
| a. UBOS b. 2011 c. 2012 | Population’s demographic characteristics, family planning efforts, maternal mortality, and infant and child mortality. a. Tobacco use b. Obesity/overweight | a. Cross-sectional b. stratified cluster sampling c. 95% d. Q & M | 11,820 | 2573 | 9,247 | a. All regions  b. Urban and Rural c. Whole country | a. Whole country b. 15-54 years |
| a. Mondo b. 2011-2012 c. 2013 | Prevalence of risk factors for NCDs.  a. Tobacco use b. Alcohol use c. Unhealthy diet d. physical inactivity  e. overweight/obesity | a. Cross-sectional b. Cluster random sampling c. 85% d. Q & M | 518 | 287 | 231 | a. Western  b. Rural c. Kasese | a. Rural Ugandan district b. > 25 years |
| a. Mayega b. 2012 c. 2012 | Socio-behavioural characteristics associated with being overweight. a. Alcohol use b. Unhealthy feeding c. physical inactivity  d. overweight/obesity e. Tobacco use | a. Cross-section b. Multistage random sampling c. 98.6% d. Q & M | 1656 | 805 | 851 | a. Eastern  b. Urban and Rural c. Iganga and Mayuge | a. people aged 35-60 years b. 35-60 years |
| a. Nuwaha b. 2012 c. 2013 | Prevalence of pre-hypertension and associated risk factors a. Tobacco use b. Alcohol use c. Unhealthy feeding d. physical inactivity  e. overweight/obesity | a. Cross-sectional b. Multistage sampling  c. 94.7% d. Q& M | 4,142 | 1,477 | 2,665 | a. Central  b. Urban and Rural  c. Mukono & Buikwe | a. Buikwe and Mukono districts b. > 18 years |
| a. Gemert  b. 2012 c. 2015 | Prevalence of chronic obstructive pulmonary disease (COPD) and related risk factors. a. Tobacco use | a. Cross-sectional b. Simple random sampling c. 97% d. questionnaire | 588 | 291 | 297 | a. Western b. Rural c. Masindi | a. rural region of Uganda b. > 30 years |
| a. Nakibuuka b.2012-2013  c.2015 | Prevalence of stroke risk factors and the associated socio-behavioural and demographic characteristics  a. overweight/Obesity | a. Cross-sectional b. Multistage stratified  c. NR d. Q & M | 5314 | 1627 | 3687 | a. Central b. Urban and Rural c. Wakiso | a. most populous district in Uganda. b. > 18 years |
| a. Kavishe b. 2012-2013 c. 2015 | Prevalence of selected NCDs and HIV infection.  a. Tobacco use b. Alcohol use c. Unhealthy feeding d. Physical inactivity e. overweight/obesity | a. Cross-sectional b. Multistage sampling c. 79% d. Q & M | 916 | 375 | 541 | a. Central  b. Urban and rural C. Entebbe | a. Southern Uganda b. > 18 years |
| a. MOH b. 2013 c. 2013 | Monitor adult use of tobacco and key tobacco control indicators a. Tobacco use | a. Cross-sectional b. multi-stage stratified cluster sampling c. 86.6% d. Questionnaire | 8,508 | 3,853 | 4,655 | a. All regions b. Rural and Urban c. Whole country | a. Whole country b. > 15 years |
| a. Schramm b. 2011-2013 c. 2016 | Prevalence of adult malnutrition and associated risk factors a. Tobacco use b. Alcohol use c. overweight/obesity | a. Cross-sectional b. NR c. 93.6% d. Q & M | 4, 986 | 2062 | 2924 | a. Northern b. Rural c. Gulu | a. post conflict area  b. > 15 years |
| a. Kirunda b. 2013 b. 2015 | Prevalence of overweight and obesity and associated factors  a. overweight and obesity | a. Cross-sectional b. Cluster simple random sampling  c. 100% d. Q & M | 1210 | 604 | 606 | a. Eastern  b. Peri-urban and rural c. Iganga and Mayuge | a. adults in peri-urban and rural Uganda b. > 18 years |
| a. Kirunda b. 2013 c. 2016 | Prevalence and associated factors of sedentary behaviour and physical inactivity. a. Physical inactivity | a. Cross-sectional b. Cluster simple random sampling  c. 100% d. Q & M | 1208 | 603 | 605 | a. Eastern  b. Peri-urban and rural c. Iganga and Mayuge | a. adults in peri-urban and rural Uganda b. > 18 years |
| a. Nalwadda  b. 2013 c. 2018 | Prevalence of alcohol consumption and of alcohol use disorder among  a. Alcohol use | a. Cross-sectional b. Simple random sampling c. 99.9% d. Questionnaire | 351 | 351 | N/A | a. Eastern b. Rural c. Kamuli | a. men in in Kamuli District,  b. > 18 years |
| a. Kwarisiima b.2013 c. 2016 | Risk factors for hypertension  a. overweight/obesity | a. Cross-sectional b. NR c. 70% d. Q & M | 65544 | 27120 | 38424 | a. Eastern and Western b. Rural c. Mbale, Tororo and Mbarara | a. Eastern and Western Uganda c. > 18 years |
| a. Ministry of health  b. 2014 c. 2016 | Prevalence of hypertension, diabetes and NCD risk factors.  a. Tobacco use b. Alcohol use c. physical inactivity  d. overweight/obesity e. Unhealthy diet | a. Cross-sectional b. Multistage sampling c. 92.2% d. Q & M | 3,987 | 1,604 | 2,383 | a. All regions  b. Urban and rural c. Whole country | a. Whole country b. 18-69 years |
| a. UBOS  b. 2016 c. 2018 | Basic demographic and health indicators.  a. Tobacco use  b. Obesity/overweight | a. Cross-sectional  b. Stratified Random sampling c. 98% d. Q & M | 23,842 | 5,336 | 18,506 | a. All regions b. Urban and Rural c. Whole country | a. Whole country b. 15-54 years |
| a. Ajayi b. NR c. 2016 | Burden of health conditions in sub-Saharan Africa and to determine the differences between urban and rural populations.  a. Tobacco use,  b. overweight/Obesity | a. Cross-sectional  b. Random sampling c. 96% d. Q & M | 497 | 239 | 258 | a. Central and Western b. Peri-Urban and Rural c. Kampala & Mbarara | a. Sub-Saharan Africa b. > 18 years |
| a. Twinamasiko  b. NR c. 2018 | Prevalence of hypertension and its association with sedentary lifestyle. a. Tobacco use,  b. Physical inactivity c. Unhealthy diet  d. overweight/obesity | a. Cross-sectional b. Multistage sampling c. NR d. Q & M | 310 | 155 | 155 | a. Western  b. Peri-urban c. Mbarara | a. Peri-urban area  b. > 35 years |

UBOS= Uganda Bureau of Statistics; NR=Not reported; Q&M = questionnaires and measurements

**Appendix 2: Prevalence (%) of tobacco use among people aged > 15 years in Uganda**

| Author (year of field work) | Sex | Urban | Rural | | Eastern | Western | Central | Northern | Karamoja | Overall | Measure of tobacco use |
| --- | --- | --- | --- | --- | --- | --- | --- | --- | --- | --- | --- |
| UBOS 2000 | M | 19.6 | 26.3 | | 15.3 | 28.6 | 24.2 | 39.8 | - | 25.2 | Current tobacco smoking |
|  | F | 0.8 | 3.8 | | 0.8 | 9.0 | 1.2 | 2.9 | - | 3.3 |  |
| UBOS 2006 | M | 15.5 | 23.0 | | 12.1 | 26.1 | 16.1 | 37.3 | 58.8 | 22.6 | Current tobacco user (all forms of tobacco) |
|  | F | 2.5 | 4.1 | | 0.4 | 4.5 | 1.4 | 8.8 | 52.2 | 3.8 |  |
| Wamala 2006 | M | - | 14.0 | | - | 14.0 | - | - | - | - | Current tobacco use (all forms of tobacco) |
|  | F | - |  |  | - |  | - | - | - | - |  |
| Kizza 2008 | M | - | - | | - | - | 12.0 | - | - | - | Current smoking |
|  | F | 0.5 | - | | - | - |  | - | - | - |  |
| UBOS 2011 | M | 8.2 | 16.4 | | 9.5 | 13.2 | 12.0 | 26.9 | 46.2 | 15.7 | Current tobacco use (all forms of tobacco) |
|  | F | 1.2 | 3.2 | | 0.5 | 3.0 | 1.5 | 2.2 | 35.6 | 2.8 |  |
| Mondo 2011 | M | - | 22.5 | | - | 22.5 | - | - | - | - | Tobacco smoking |
|  | F | - | 15.5 | | - | 15.5 | - | - | - | - |  |
| Mayega 2012 | M | - | | - | 5.9 | - | - | - | - | - | Current tobacco use (all forms of tobacco) |
|  | F | - | | - |  | - | - | - | - | - |  |
| Nuwaha 2012 | M | 14.6 | | | - | - | 6.4 | - | - | - | Currently smoking |
|  | F | 2.0 | | | - | - |  | - | - | - |  |
| Germent 2012 | M | - | 34.4 | | - | 34.4 |  | - | - | - | Current smokers |
|  | F | - | 7.4 | | - | 7.4 |  | - | - | - |  |
| Kavishe 2012 | M | 17.7 | 17.4 | | - | - | - | - | - | - | Current smoking |
|  | F | 1.6 | 2.1 | | - | - | - |  |  |  |  |
| Schramm 2013 | M | - | 32.0 | | - | - | - | 32.0 | - | - | Smoking daily. |
|  | F | - | 0.5 | | - | - | - | 0.5 | - | - |  |
| MOH 2013 | M | 9.9 | 10.4 | | - | - | - | - | - | 11.6 | Current tobacco use (all forms of tobacco) |
|  | F | 1.7 | 1.8 | | - | - | - | - | - | 4.6 |  |
| MOH 2014 | M | 11.3 | 15.2 | | 7.0 | 9.8 | 5.4 | 16.8 | - | 18.0 | Current tobacco use (all forms of tobacco) |
|  | F | 0.9 | 3.0 | |  |  |  |  | - | 5.0 |  |
| UBOS 2016 | M | 7.5 | 10.0 | | 5.0 | 9.9 | 8.3 | 17.5 | 5.6 | 10.1 | Current tobacco use ( all forms of tobacco) |
|  | F | 0.6 | 0.9 | | 0.5 | 1.1 | 0.7 | 0.9 | 0.3 | 0.8 |  |
| Ajayi NR | M | 6% | 13% | | - | 13.0 | 6.0 |  |  |  | Ever smoked |
|  | F |  |  |  | - |  |  | - | - | - |  |
| Twinamasiko NR | M | 27.8 | - | | - | 27.8 | - | - | - | - | Current smoking |
|  | F |  | - | | - |  | - | - | - | - |  |

M=Male; F= Female; NR=Not reported; Figures for Northern Uganda exclude Karamoja sub-region

**Appendix 3: Prevalence (%) of alcohol use among people aged >15 years in Uganda**

| Author and year of field work | Sex | Urban | Rural | Eastern | Western | Central | Northern | Overall | Measure and definition of harmful use of alcohol |
| --- | --- | --- | --- | --- | --- | --- | --- | --- | --- |
| UBOS 2000 | M | 41.0 | 45.7 | 46.8 | 45.6 | 39.2 | 54.0 | 45.0 | Consumption of alcohol in the past 30 days |
|  | F | 23.6 | 24.1 | 27.1 | 17.2 | 21.3 | 34.7 | 24 |  |
| Wamala 2006 | M | **-** | 43.3% | **-** | 43.3% | **-** | **-** | **-** | Current alcohol users |
|  | F | **-** |  | **-** |  | **-** | **-** | **-** |  |
| Erlt 2010 | M | 46 | | - | - | - | 46 | **-** | Harmful use of alcohol using AUDIT score > 8 |
|  | F | 1 | | - | - | - | 1 | **-** |  |
| Mondo 2011 | M | **-** | 4.1 | - | 4.1 | **-** | **-** | **-** | Harmful use of alcohol or excessive drinking using WHO STEPwise criteria |
|  | F | **-** | 1.2 | - | 1.2 | **-** | **-** | **-** |  |
| Nuwaha 2012 | M | 36.6 | | **-** | **-** | 25.8 | **-** | **-** | Current alcohol consumption |
|  | F | 19.8 | | **-** | **-** |  | **-** | **-** |  |
| Kavishe 2012 | M | 20.0 | 11.9 | - | - | - | - | **-** | Problem drinking (measured as AUDIT score ≥ 8) |
|  | F | 2.2 | 5.5 | - | - | - | - | **-** |  |
| Mayega 2012 | M | - | - | 4.7 | - | - | - | **-** | Harmful use of alcohol; consuming > 60g  in the 1 month preceding the survey |
|  | F | - | - |  | - | - | - | **-** |  |
| Schramm 2013 | M | - | 6.4 | - | - | - | 6.4 | **-** | Consumed alcohol 5-7 days per week |
|  | F | - | 1.1 | - | - | - | 1.1 | **-** |  |
| Nalwadda 2013 | M | - | 4.1 | 4.1 | - | - | - | - | Harmful/dependant/hazardous use of alcohol (AUDIT positive: AUDIT score of > 8 ) |
|  | F | **-** | - | - | - | - | - | - |  |
| MOH 2014 | M | 17.1 | 19.6 | 13.7 | 21.4 | 18.5 | 23.2 | 25.9 | Medium- to high-end alcohol users (sub-analysis of Kabwana 2016). |
|  | F |  |  |  |  |  |  | 14.3 |  |
| Twinamasiko NR | M | 8.4% | - | - | - | 8.4% | - | - | Harmful use of alcohol using WHO STEPwise criteria (> 5 days per week) |
|  | F |  | **-** | **-** | **-** |  | **-** | **-** |  |

M=Male; F= Female; NR=Not reported; AUDIT= Alcohol Use Disorder Identification; WHO=World Health Organisation

**Appendix 4: Prevalence (%) of unhealthy diet among people aged >15 years in Uganda**

| Author and year of field work | Sex | Urban | Rural | Eastern | Western | Central | Northern | Overall | Measure and definition of Unhealthy diet |
| --- | --- | --- | --- | --- | --- | --- | --- | --- | --- |
| UBOS 2006 | M | - | - | - | - | - | - | - | Did not consume Vitamin A -rich fruits/vegetables in the past 24 hours |
|  | F | 47.9 | 47.6 | 36.4 | 54.8 | 59.7 | 38.4 | 47.6 |  |
| Mondo 2011 | M | - | 91 & 98.8 | - | 92.8 & 98.8 | - | - | - | Less than 5 serving of fruits and vegetables per week |
|  | F | - | 94.3 & 98.9 | - |  | - | - | - |  |
| Mayega 2012 | M | - | - | 89.8% | - | - | - | - | foods eaten in 7 days prior survey (low-moderate dietary diversity) |
|  | F | - | - |  | - | - | - | - |  |
| Nuwaha 2012 | M | 60.4 & 48.7 | | - | - | 59.1 & 45.8 | - | - | < 7 times of consuming fruits and vegetables in the previous week. |
|  | F | 58.6 & 44.3 | | - | - |  | - | - |  |
| Kavishe 2012 | M | 41.8 | 70.0 | - | - | 41.8 | - | - | Eat fewer than one serving of fruits or vegetables per day |
|  | F | 46.5 | 63.5 | - | - | 46.6 | - | - |  |
| MOH 2014 | M | 88.4 | | - | - | - | - | 87.8 | Less than 5 servings of fruits and vegetables per day. |
|  | F | 87.3 | | - | - | - | - |  |  |
| Twinamasiko NR | M | - | 60.6 | - | 60.6 | - | - | - | Eat vegetables less than 3 days per week. |
|  | F | - |  | - |  | - | - | - |  |

M=Male; F= Female; NR=Not reported

**Appendix 5: Prevalence (%) of physical inactivity among people aged >15 years in Uganda**

| Author and year of field work | Sex | Urban | Rural | Eastern | Western | Central | Northern | Overall | Measure and definition of physical inactivity |
| --- | --- | --- | --- | --- | --- | --- | --- | --- | --- |
| Wamala 2006 | M | - | - | - | 18 | - | - | - | Sedentary occupation |
|  | F | - | - | - |  | - | - | - |  |
| Mondo 2011 | M | - | 48.0 |  | 51.0 | - | - | - | No activity at workplace, during recreation, travelling and resting time |
|  | F | - | 50.0 |  |  | - | - | - |  |
| Nuwaha 2012 | M | 13.8 | | - | - | 16.3 |  |  | Infrequent/none: < 3 times of walking, riding, exercises, sports, manual work etc.) per week |
|  | F | 17.7 | | - | - |  | - | - |  |
| Kavishe 2012 | M | 59.5 | 72.5 | - | - | - | - | - | No vigorous activity per week |
|  | F | 81.9 | 91.9 | - | - | - | - | - |  |
| Mayega 2012 | M | - | - | 14.6 |  |  |  |  | No days of vigorous physical activity per week |
|  | F | - | - |  |  |  |  |  |  |
| Kirunda 2013 | M | 28.5 | - | 37.6 | - | - | - | - | Physically inactive (daily average of < 7500 steps taken |
|  | F | 46.6 | - |  | - | - | - | - |  |
| MOH 2014 | M | 8.9 | 4.6 | 6.8 | 3.4 | 8.1 | 3.3 | 3.7 | Not meeting WHO recommendations of PA for Health |
|  | F |  |  |  |  |  |  | 4.9 |  |
| Twinamasiko NR | - | 65.2 | - | - | 65.2 | - | - | - | Sedentary: no activity lasting for more than 10 minutes at work style or recreation or leisure |
|  | - |  | - | - |  | - | - | - |  |

*M=Male; F= Female; NR=Not reported; PA=Physical Activity
WHO recommendations for PA=at least 150 minutes of moderate-intensity PA or; 75 minutes of vigorous-intensity PA or an equivalent combination of moderate and vigorous intensity PA achieving at least 600 MET-minutes.[13]. METs = metabolic equivalents.*

**Appendix 6: Proportion (%) of overweight [obesity] among people aged > 15 years in Uganda**

| Author & year of field work | Sex | Urban | Rural | Eastern | Western | Central | Northern | Overall |
| --- | --- | --- | --- | --- | --- | --- | --- | --- |
| UBOS 2000 | M | - | - | - | - | - | - | - |
|  | F | 30.2 | 10.6 | 9.0 | 14.8 | 21.5 | 5.1 | 13.8 |
| Baalwa 2004 | M | 3.3 [1.8] | | 10.6 [0.0] | - | 10.4 [4.4] | - | 10.4 [2.3] |
|  | F | 17.4 [2.9] | |  | - |  | - |  |
| Kikafunda 2002 | M | - | - | - | - | 8.6 [0.0] | - | - |
|  | F | - | - | - | - |  | - | - |
| Wamala 2006 | M | - | - | - | 32.0 [8.0] | - | - | - |
|  | F | - | - | - |  | - | - | - |
| UBOS 2006 | M | - | - | - | - | - | - | - |
|  | F | 21.5[12.2] | 10.5[2.4] | 6.5 [1.6] | 17.2 [4.5] | 18.4 [8.1] | 5.4 [1.0] | 12.4 [4.1] |
| UBOS 2011 | M | 9.8 [1.7] | 2.3 [0.3] | 2.0 [0.1] | 4.6 [0.8] | 5.7 [1.0] | 1.8 [0.0] | 4.0 [0.6] |
|  | F | 25.5 [9.5] | 11.6 [2.7] | 10.9 [1.7] | 18.5[4.9] | 20.4 [7.6] | 5.5 [0.4] | 14.6 [4.2] |
| Mondo 2011 | M | - | 14.7 [4.9] | - | 15.6 [6.7] | - | - | - |
|  | F | - | 16.7 [9.0] | - |  | - | - | - |
| Nuwaha 2012 | M | 6.9 [1.5] | | - | - | 14.2 [6.0] | - | - |
|  | F | 18.3 [8.5] | | - | - |  | - | - |
| Kirunda 2013^*^ | M | 23.8 [17.8] | 15.8 [3.9] | 12.4 [2.0] | - | - | - | - |
|  | F |  |  | 23.1 [12.7] | - | - | - |  |
| Schramm 2013 | M | - | 1.5 [0.0] | - | - | - | 1.5 [0.0] | - |
|  | F | - | 6.8 [0.8] | - | - | - | 6.8 [0.8] | - |
| Kavishe 2012 | M | 9.1 [2.6] | 8.5 [0.7] | - | - | - | - | - |
|  | F | 24.8 [18.2] | 30[11.4] | - | - | - | - | - |
| Mayega 2012 | M | 7.5 [2.2 | | 12.6 [5.3] | - | - | - | - |
|  | F | 16.9 [8.2] | |  | - | - | - | - |
| Nakibuuka 2012-2013^*^ | M | 42% | 22% | - | - | - | - | - |
|  | F |  |  | - | - | - | - | - |
| Kwarisima 2013 | M | - | 8.0 [1.0] | - | - | - | - | 14.0 [3.0] |
|  | F | - | 18.0 [5.0] | - | - | - | - |  |
| MOH 2014 | M | 15.8 [3.1] | 8.1 [1.5] | - | - | - | - | 9.5 [1.8] |
|  | F | 28.8 [15.6] | 17.2 [5.5] | - | - | - | - | 19.5 [7.5] |
| UBOS 2016 | M | 13.1 [3.2] | 5.5 [0.6] | 5.9 [0.5] | 7.2 [0.8] | 12.8 [2.8] | 3.0 [0.7] | 7.7 [1.2] |
|  | F | 21.8 [12.5] | 14.6 [5.3] | 13.6 [3.5] | 20.6[6.7] | 22.4 [14.6] | 8.5 [1.5] | 16.5 [7.2] |
| Ajayi NR^*^ | M | 13.7 [3.1] | 35.5 [2.4] | - | 36 [10] | 24 [14] | - | - |
|  | F | 32.7 [23.1] | 42 [19] | - |  |  | - | - |
| Twinamasiko NR | M | - | - | - | [46] | - | - | - |
|  | F | - | - | - |  | - | - | - |

NR=not reported; Northern proportions exclude Karamoja region

**Appendix 7: Factors associated with Overweight (>25kg/m2) in Uganda**

| **Risk Factor** | **Author** | **Odds Ratio (95% CI)** |
| --- | --- | --- |
| Sex (women vs men) | Baalwa 2010 | 4.81 [2.76-8.36] |
|  | Mayega 2012 | 3.7 [2.69–5.08] |
|  | Nakibuuka 2015 | 3.68 [2.6–5.1] for rural 4.16 [3.5–4.9] for urban |
|  | Ajayi 2016 | 2.12 [1.12-4.01] |
|  | Kirunda 2015 | 4.3 [3.2–5.9] |
| Residing in Urban Vs rural | UBOS 2011 ( in Ture 2013, Neupane 2016 & Madise 2017 ) | 3.20 [2.41–3.07] |
|  | Mayega 2012 | 2.1 [1.46–3.01] |
|  | Kirunda 2015 | 2.6 [1.9–3.6] |
| Rich (high SES) vs poor (low SES) Or  Fifth compared to first SES quintile | UBOS 2011 (in Turi 2013 & Neupane 2016) | 4.68 [3.77–5.82] |
|  | Nakibuuka 2015 | 1.79 [1.5–2.2] for urban |
|  | Kirunda 2015 | 3^rd^ SES quintile 2.8 [1.7-4.6] 4^th^ SES quintile 2.5 [1.5-4.2] 5^th^ SES quintile 2.7 [1.6-4.4] |
|  | Mayega 2012 | 4.1[2.40–6.98] |
| Increasing age vs 18–24 years | Kirunda 2015 | 35–44 years 3.1[1.8–5.3]  45–54 years 4.1 [2.3–7.3]  55–64 years 2.6 [1.4–5.0]  ≥ 65 years 3.1 [1.6–6.0] |
|  | Mayega 2012 | 50-52 years 1.8 [1.1–2.79] 45-49 years 1.6 [1.1-2.4] |
|  | Nakibuuka 2015 | 5.87 [3.9–8.9] |
| Family history of diabetes or having diabetes | Mayega 2012 | 1.5[1.02–2.22] |
|  | Nakibuuka | 1.38 [1.1–1.8] |
| High vs low education | UBOS 2011 (in Neupane 2016)  Baalwa 2010 | 2.18 [1.79–2.65]  11.47 [1.51-87.32] |
| Being married Vs never married  divorced/separated/widowed vs never married | Ajayi 2016 | 3.93 [1.60- 9.64] |
|  | Ajayi 2016 | 3.87 [1.10-13.56] |
| Alcohol consumption  Smoking  Commuting to school by taxi or private vehicle | Baalwa 2010 | 4.96 [1.75-14.01]  13.56 [3.94-46.66] |
|  |  | 6.41 [2.04-20.11] |
